# Tape Worm

**Published:** 1852-09

**Authors:** 


*Tape Worm.*

SINCE the article of Dr. Castleman, in the present No. of the Journal, was in type, our attention has been called to the following notice of a West Indian remedy for the expulsion of this species of parasite:

At one of the late meetings of the Medical Society of Bordeaux, M. Brunet communicated to the society several cases of tape-worm, where he had succeeded in causing the expulsion of the parasite by means of a paste made of pumpkin seeds, stating that he had been told of the remedy by the captain of a ship. Since that period an article published in the *Journal Universel* of 1820, was discovered, where M. Mongeny, a physician of the island of Cuba, says:—"I used to give to patients affected with tænia three ounces of a paste made with fresh pumpkin seeds, and afterwards six ounces of honey, in three doses: the first an hour after the injestion of the paste, and the others at the same intervals. Six or seven hours afterwards the tænia was generally expelled, and this remedy has succeeded n cases which had resisted all the means generally employed. The worm is rejected, not in fragments, but twisted upon itself; and where two parasites had existed, they were wholly and simultaneously voided.*

The action of the pumpkin seeds and honey together seems to be somewhat different from that of the honey alone as observed by Dr. Castleman; the one expelling the parasite whole, the other in pieces. J.

* Vide London Lancet, August, 1852, page 149.

